# HSV-1 Genome Subnuclear Positioning and Associations with Host-Cell PML-NBs and Centromeres Regulate LAT Locus Transcription during Latency in Neurons

**DOI:** 10.1371/journal.ppat.1002852

**Published:** 2012-08-09

**Authors:** Frédéric Catez, Christel Picard, Kathrin Held, Sylvain Gross, Antoine Rousseau, Diethilde Theil, Nancy Sawtell, Marc Labetoulle, Patrick Lomonte

**Affiliations:** 1 Virus and Centromere team, Centre de Génétique et de Physiologie Moléculaire et Cellulaire CNRS, UMR5534, Villeurbanne, France; 2 Université de Lyon 1, Lyon, France; 3 Institut de Virologie Moléculaire et Structurale, CNRS-UPR-3296, Gif-sur-Yvette, France; 4 Department of Clinical Neurosciences, Ludwig Maximilian University, Munich, Germany; 5 Laboratoire d'excellence, LabEX DEVweCAN, Lyon, France; 6 Division of Infectious Diseases, Cincinnati Children's Hospital Medical Center, Cincinnati, Ohio, United States of America; 7 Ophtalmology Department, Hopital Bicêtre, APHP, Université Paris-Sud, Kremlin-Bicêtre, France; University of Glasgow, United Kingdom

## Abstract

Major human pathologies are caused by nuclear replicative viruses establishing life-long latent infection in their host. During latency the genomes of these viruses are intimately interacting with the cell nucleus environment. A hallmark of herpes simplex virus type 1 (HSV-1) latency establishment is the shutdown of lytic genes expression and the concomitant induction of the latency associated (LAT) transcripts. Although the setting up and the maintenance of the latent genetic program is most likely dependent on a subtle interplay between viral and nuclear factors, this remains uninvestigated. Combining the use of *in situ* fluorescent-based approaches and high-resolution microscopic analysis, we show that HSV-1 genomes adopt specific nuclear patterns in sensory neurons of latently infected mice (28 days post-inoculation, d.p.i.). Latent HSV-1 genomes display two major patterns, called “Single” and “Multiple”, which associate with centromeres, and with promyelocytic leukemia nuclear bodies (PML-NBs) as viral DNA-containing PML-NBs (DCP-NBs). 3D-image reconstruction of DCP-NBs shows that PML forms a shell around viral genomes and associated Daxx and ATRX, two PML partners within PML-NBs. During latency establishment (6 d.p.i.), infected mouse TGs display, at the level of the whole TG and in individual cells, a substantial increase of PML amount consistent with the interferon-mediated antiviral role of PML. “Single” and “Multiple” patterns are reminiscent of low and high-viral genome copy-containing neurons. We show that LAT expression is significantly favored within the “Multiple” pattern, which underlines a heterogeneity of LAT expression dependent on the viral genome copy number, pattern acquisition, and association with nuclear domains. Infection of PML-knockout mice demonstrates that PML/PML-NBs are involved in virus nuclear pattern acquisition, and negatively regulate the expression of the LAT. This study demonstrates that nuclear domains including PML-NBs and centromeres are functionally involved in the control of HSV-1 latency, and represent a key level of host/virus interaction.

## Author Summary

After an initial lytic infection, many viruses establish a lifelong latent infection that hides them from the host immune system activity until reactivation. To understand the resurgence of the associated diseases, it is indispensable to acquire a better knowledge of the different mechanisms involved in the antiviral defense. During latency, viral genomes of nuclear-replicative viruses, such as herpes simplex virus type 1 (HSV-1), are stored in the nucleus of host cells in a non-integrated form. Latency establishment is associated with a drastic change in HSV-1 gene expression program that is maintained until reactivation occurs. The last two decades of research has revealed that the functional organization of the cell nucleus, so-called nuclear architecture, is a major factor of regulation of cellular genes expression. Nonetheless, the role of nuclear architecture on HSV-1 gene expression has been widely overlooked. Here we describe that the genome of HSV-1 selectively interacts with two major nuclear structures, the promyelocytic nuclear bodies (PMLNBs or ND10) and the centromeres. We provide evidence supporting that these nuclear domains directly influence the behavior of latent viral genomes and their transcriptional activity. Overall, this study demonstrates that nuclear architecture is a major parameter driving the highly complex HSV-1 latency process.

## Introduction

Herpes simplex virus type 1 (HSV-1), a major human pathogen, is a persistent human neurotropic virus and a model of long-term interaction between a host cell and a parasite. HSV-1 establishes a long-term latent infection in neurons of the trigeminal (or Gasserian) ganglia (TG) of the peripheral nervous system, from which it reactivates periodically to replicate and spread [1]. The establishment of latency is dependent on a sequence of physiological and molecular events involving the host immune system, the cellular antiviral response, and the ability of the virus to initiate a latent gene expression program.

Latent HSV-1 dsDNA genomes localize in the nucleus of the host neuron where they remain as multi-copy chromatinized episomes, which do not integrate into the host-cell genome [2], [3]. During latency, HSV-1 lytic gene expression is strongly repressed; although some lytic transcripts could be detected at low level, by highly sensitive techniques [4]–[6]. The latency-associated transcript (LAT) locus is the only gene to be highly expressed throughout the persistent stage, from establishing latency to reactivation [7]. LAT is a noncoding RNA, synthesized as an 8.3-kb polyadenylated, unstable primary transcript, and is rapidly processed into a stable 2-kb intron lariat and several microRNAs [8]–[11]. LAT expression has been linked to several aspects of the latency process, including neuron survival, viral genome chromatin status, lytic gene expression, number of latently infected neurons, and efficiency of reactivation in animal models [2], [3], [10], [12]–[17]. Although LAT appears to regulate latency and reactivation, several studies have shown that LAT is probably expressed only in a subset of latently infected neurons, implying that latency is intrinsically a heterogeneous event [18]–[23]. The heterogeneity of HSV-1 latency has also been observed at the level of the viral genome copy number in individual neurons, which has been directly correlated with reactivation probability, suggesting that it is a functionally significant parameter [19], [20], [24]. How these variable parameters impact on the biology of the latent virus and the reactivation process remains unclear. Moreover, host-cell factors and the cellular environment can be anticipated to also account for the variability of latency and for determining the ability of HSV-1 to reactivate. Therefore, the study of latency requires experimental approaches in which the heterogeneity can be fully assessed with regard to viral genome features, viral gene expression, and host-cell nuclear components. In situ fluorescence-based strategies offer such a possibility, through a multi-parametric reading of a cell population at the single-cell level.

The mammalian cell nucleus is a highly organized compartment containing the chromosomes and several nuclear domains, which reflect the various molecular activities taking place in the nucleus. Numerous studies reported that the position of a gene within the nucleus is correlated with its transcriptional status [25], [26]. The predetermined nuclear positions of genetic loci within the nuclear architecture are key determinants of gene expression, together with transcription factors and epigenetic chromatin modifications [25], [27], [28]. Nuclear structures known to influence gene expression include the nuclear envelope, telomeres, centromeres and pericentromeres, and nuclear domains such as promyelocytic leukemia (PML) nuclear bodies (NBs, also called ND10), transcription factories, polycomb group complexes, and the nucleolus [26], [29]–[34]. Among these nuclear domains, PML-NBs are proteinaceous structures that reorganize in response to various cellular stressors [33], [35], [36]. PML-NBs provide a nuclear environment that can be associated with transcription of cellular genes [32], [37], [38]. However, PML-NBs contain repressor proteins such as HP1, ATRX, and hDaxx [39], [40], which have an inhibitory effect on transcription and replication of RNA and DNA viruses, supporting the silencing activity of PML-NBs [41], [42]. In cultured infected cells, the association of PML-NB with genomes of several viruses, including HSV-1, has led to the hypothesis that PML-NBs may operate as a nuclear relay for innate host-cell defense mechanisms, blocking replicative infection by creating an environment unfavorable for viral gene expression [32], [42]–[50]. However, how nuclear domains impact *in vivo* on the biology of persistent viruses such as HSV-1 and whether they may intervene in the latency process, in particular in the acquisition of essential parameters involved in latency maintenance and reactivation, is currently unknown.

In this study, we took advantage of a physiologically well-characterized mouse model of HSV-1 infection, to develop an efficient fluorescent in situ hybridization (FISH) approach for detecting HSV-1 genomes during latency in neurons from infected mouse TG. Using a high-resolution visualization technique, we described the intra-nuclear distribution of the latent HSV-1 genome in neurons, and correlated HSV-1 patterns with LAT expression. We found that HSV-1 genomes were non-randomly associated with two nuclear domains, PML-NBs and centromeres. Using infected PML knockout (KO) mice, we showed that PML/PML-NBs influence viral genome distribution and negatively regulate expression of LAT. Finally, we demonstrated that HSV-1 genomes associated with PML-NBs or centromeres were negative for the expression of LAT.

## Results

### *In situ* detection of latent HSV-1 genomes by FISH

**Figure 1:**
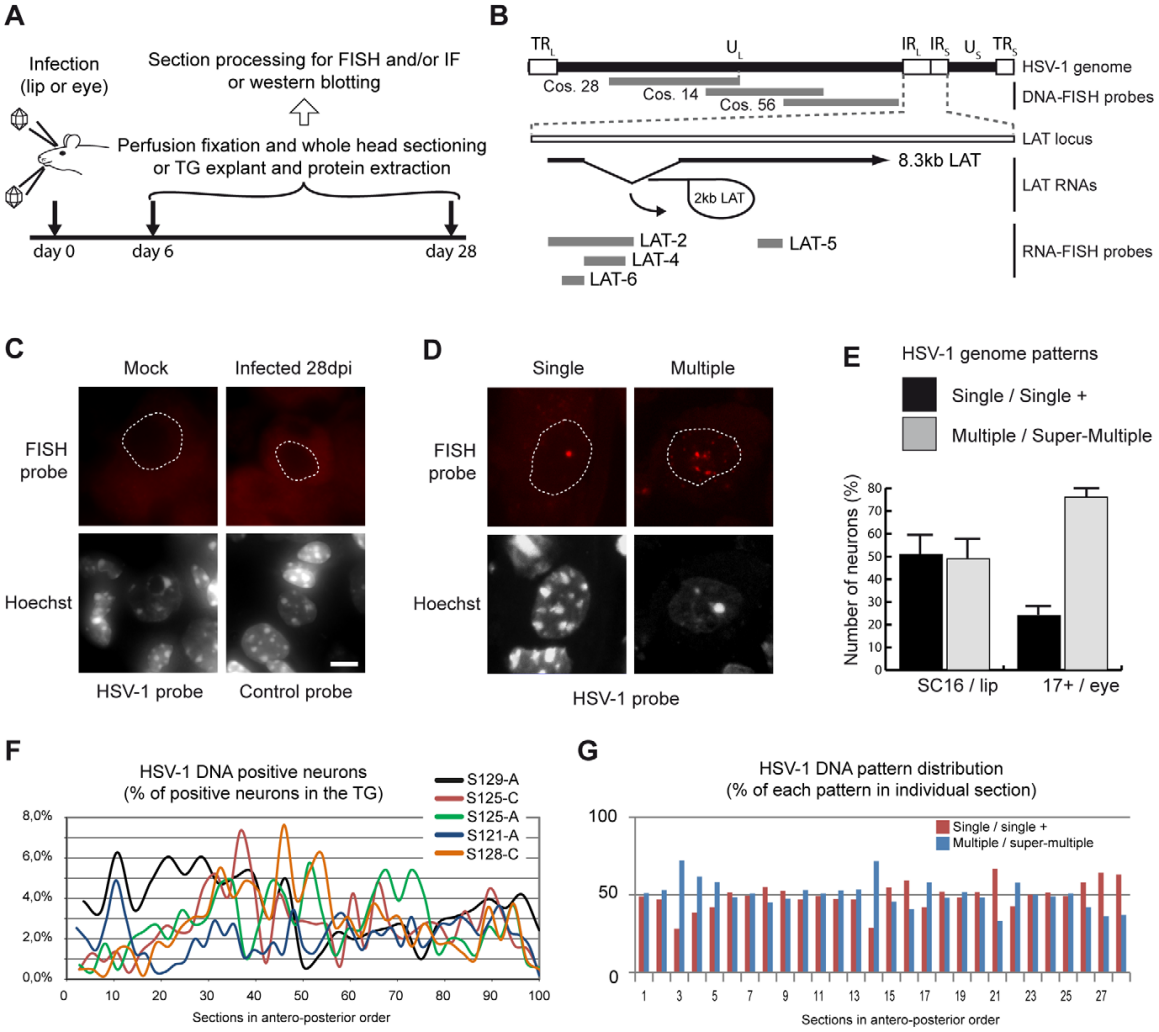
*In situ* detection of the HSV-1 genome in mouse TG sections. (A) Models of latent infection. (B) Schematic representation of the HSV-1 genome and the LAT locus. The RNA- and DNA-FISH probes used in this study are indicated in grey. (C) Control DNA-FISH experiments were performed on mock-infected mouse tissue by using an HSV-1-specific probe and on HSV-1-infected mouse tissue by using an empty cosmid vector (Cos64) probe. Stained tissue sections were imaged by wide-field microscopy. Dashed lines indicate the position of the nucleus. Scale bar = 5 µm. (D) Illustration of the main latent HSV-1 genome pattern. DNA-FISH was performed on TG sections obtained from infected mice 28 d.p.i. as in (A), using a mix of HSV-1 cosmids 14, 28, and 56 as indicated in (B). Stained sections were imaged by wide-field microscopy. Dashed lines indicate position of the nucleus. Scale bar = 5 µm. (E) Quantification of HSV-1 genome patterns during latency. Two groups of mice were infected according to the two models presented in (A). For SC16/lip-infected mice, *n* = 6 (4671 infected neurons); for 17syn+/eye mice, *n* = 4 (1000 infected neurons). Bars show the standard error of the mean. (F) Distribution of latently infected neurons along the TG. The data obtained in (E) from five SC16/lip-infected mice were plotted as total number of HSV-1 genome-positive neurons per section. (G) Distribution of the single/single+ and multiple/super-multiple patterns along the TG. Data from one mouse shown in (F) is shown as example.

The lack of an efficient *in situ* detection method of viral genomes has been a major technical limitation to the study of herpes virus infection and disease both in animal models and human samples. Detection of HSV-1 genomes by FISH in latently infected mouse tissues has remained unsuccessful despite attempts of many groups [51]. To determine the intra-nuclear organization of the multiple copies of HSV-1 and its influence HSV-1 gene expression, we developed a DNA-FISH protocol and applied it to an established lip-inoculation mouse model in which HSV-1 establishes significant latency in the TG (Figure 1A; [52]). Infected and mock-infected mice were sacrificed at 28 d.p.i., a time point at which HSV-1 latency is known to be fully established [51], and the TGs were cryo-sectioned. Our FISH protocol efficiently detected latent HSV-1 genomes in mouse neuronal tissues (Figure 1D; see Materials and Methods for details). The DNA-FISH probes recognized a 90 kb region of the viral genome, excluding the LAT locus (named hereafter “HSV-1 genome probes,” Figure 1B). Importantly, our protocol did not include a signal amplification procedure and thus is well suited for the study of intra-nuclear organization by high-resolution microscopy. Signal specificity was assessed through several control experiments, including FISH analysis of mock-infected mice, FISH with control probes without HSV-1 sequences (Figure 1C), and a comparison between our probe and a commercially available probe (Figure S1).

In TG sections from infected mice sacrificed at 28 d.p.i., the FISH signal for HSV-1 DNA was observed only in the nuclei of neurons, where it appeared as a dotted pattern comprising spots of various numbers, sizes, and intensities (Figure 1D and S1). The presence of the HSV-1 genome in neurons and not in satellite cells is consistent with the results of previous *in situ* PCR and single-cell PCR studies [20], [24]. Two main intra-nuclear patterns were observed: a single, bright, round spot (termed “single”) and numerous spots of non-uniform size and shape (termed “multiple”) (Figure 1D–E). Both patterns were observed in different inoculation models (lip, eye, and whisker pads), animals (mice of different inbred strains and rabbits), and viral strains (SC16, 17syn+, KOS/M, and McKrae), indicating that they are characteristic of neurons latently infected with HSV-1. The occurrence of each pattern was variable (Figure 1E), suggesting that the strain of virus and or the route of infection may affect how the viral genome accumulates in neurons. In addition to these two primary patterns, a single spot accompanied by one or two smaller spots (termed “single+”) and a multiple pattern that filled the nucleus (termed “super-multiple”) were less frequently observed (Figure S1). The presence of multiple genome spots in a large proportion of infected neurons is consistent with the results of earlier single-cell quantitative PCR (qPCR) analyzes [19], [20]. We further confirmed that the sizes of the spots observed by FISH were consistent with the presence of several copies per spot (Table 1). The spot in the single pattern was 0.80±0.14 µm wide (*n* = 48), a size similar to that of *in vitro*-induced quiescent genomes [50], which were estimated by qPCR to contain four to five copies of the genome. The spots of the multiple pattern varied from 0.40 to 3 µm in diameter (more in the case of large aggregates). We measured the sizes of individual FISH-detected HSV-1 genomes in *in vitro*-infected cells to define a reference. Single-copy parental genomes entering the nucleus appeared as spots that were 0.51±0.08 µm wide (*n* = 76), which was similar to the width of isolated spots within the multiple pattern (0.44±0.07 µm; *n* = 51), indicating that these spots may represent single genomes. Based on this analysis, it can be predicted that the single-spot pattern contains more than one copy of the genome and that in the multiple-spot pattern, the genome can be either isolated or aggregated.

**Table 1:** Estimation of the number of genome copy per FISH spot.

	In vitro infected cells	Latent genome	
	Individual parental genome	“Single” pattern	Smaller spots in the “Multiple” pattern
**Size of the spot (µm)**	0.51+/−0.08	0.80+/−0.14	0.44+/−0.07
	n = 76	n = 48	n = 51
**Estimated number of HSV-1 genome per spot**	1	2 to 5	1

The diameter of FISH spots was measured using Metamorph software at the equatorial plan of each spot. *In vitro* HSV-1 infected cells fixed at 2 hours post infection were used as a reference. At this time of infection, the genome replication has not started and single spots represent single copy of the genome. Note that the difference of chromatin status of lytic vs latent genomes prevents any precise calibration of the spot size/copy number relationship to be established.

We next used serial sectioning to explore whether HSV-1 established latency with a topographical preference within the TG. Neurons shown by FISH to be positive for HSV-1 were distributed all along the TG (one of the three sections analyzed), without any enrichment along the antero-posterior axis (Figure 1F). Similarly, the frequencies of the single and multiple patterns were equivalent throughout the TG (one mouse is shown as an example in Figure 1G). The frequency varied from section to section, and no reproducible pattern could be detected in a group of six mice.

Overall, these results show that the HSV-1 latent genome in mouse neuronal tissues can be detected by FISH, with sufficient efficiency and quality for the analysis of intra-nuclear distribution. During latency in neuron nuclei, the HSV-1 genome is present as multiple copies, as shown previously using other methods [15], and adopts a non-random intra-nuclear organization.

### LAT expression is linked to a specific nuclear HSV-1 genome pattern

**Figure 2:**
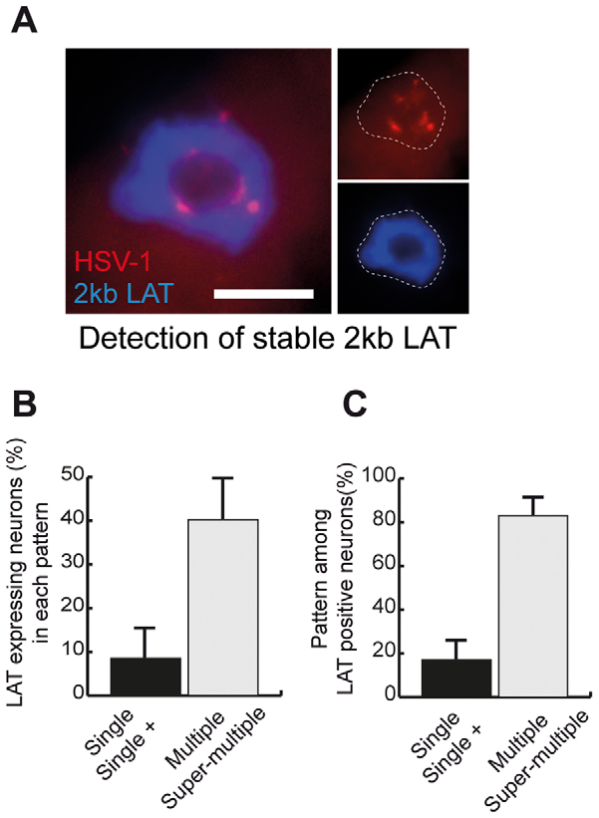
LAT expression correlates with HSV-1 genome pattern. (A) TG sections obtained from SC16/lip-infected mice at 28 d.p.i. were processed for RNA-FISH using a 2-kb LAT RNA-FISH probe and an HSV-1 DNA-FISH probe as in Figure 1D. The dotted lines outline the nucleus. Each labeling is shown as separated channels on the right panel. Wide-field imaging. Scale bar = 5 µm. (B) TG sections obtained from three mice at 28 d.p.i. were processed as in (A). The 2-kb LAT RNA-FISH signal and DNA-FISH pattern were quantified and plotted as the fraction of LAT+ neurons among neurons with the various patterns. (C) Same data set as in (B), plotted as the fraction of each pattern in LAT+ neurons. Bars show the standard error of the mean.

Data from several groups suggest that LAT is expressed in a fraction of infected neurons during latency. We set up a dual RNA/DNA-FISH assay based on tyramide signal amplification (TSA) technology to co-detect LAT transcripts and HSV-1 genomes (Figure 1B and 2A). We challenged the sensitivity of our RNA FISH method by using up to 20 times the amount of probe (1000 ng/assay instead of 50 ng) and increasing the TSA time. We failed to detect neurons with weak LAT signals, indicating that our test efficiently detected LAT-expressing neurons. In mice at 28 d.p.i., 18 to 31% of the HSV-1 DNA-containing neurons were positive for the 2-kb LAT RNA, thus confirming the results previously obtained by different approaches [18], [20]. Notably, fewer than 10% of neurons with a single pattern were positive for 2-kb LAT (Figure 2B). In contrast, 40.3±9.5% of the multiple-pattern neurons expressed LAT, suggesting that the multiple pattern reflects conditions favorable for LAT transcription (Figure 2B). A reciprocal analysis showed that 83.0% of LAT-positive neurons contained the HSV-1 genome in a multiple pattern (Figure 2C). This suggests that the organization of the HSV-1 genome in a multiple pattern is necessary, but not sufficient, to support LAT transcription. These data demonstrate that transcription of the LAT locus is linked to the intra-nuclear pattern of the viral genome.

### Latent HSV-1 genomes co-localize with centromeres in neurons

**Figure 3:**
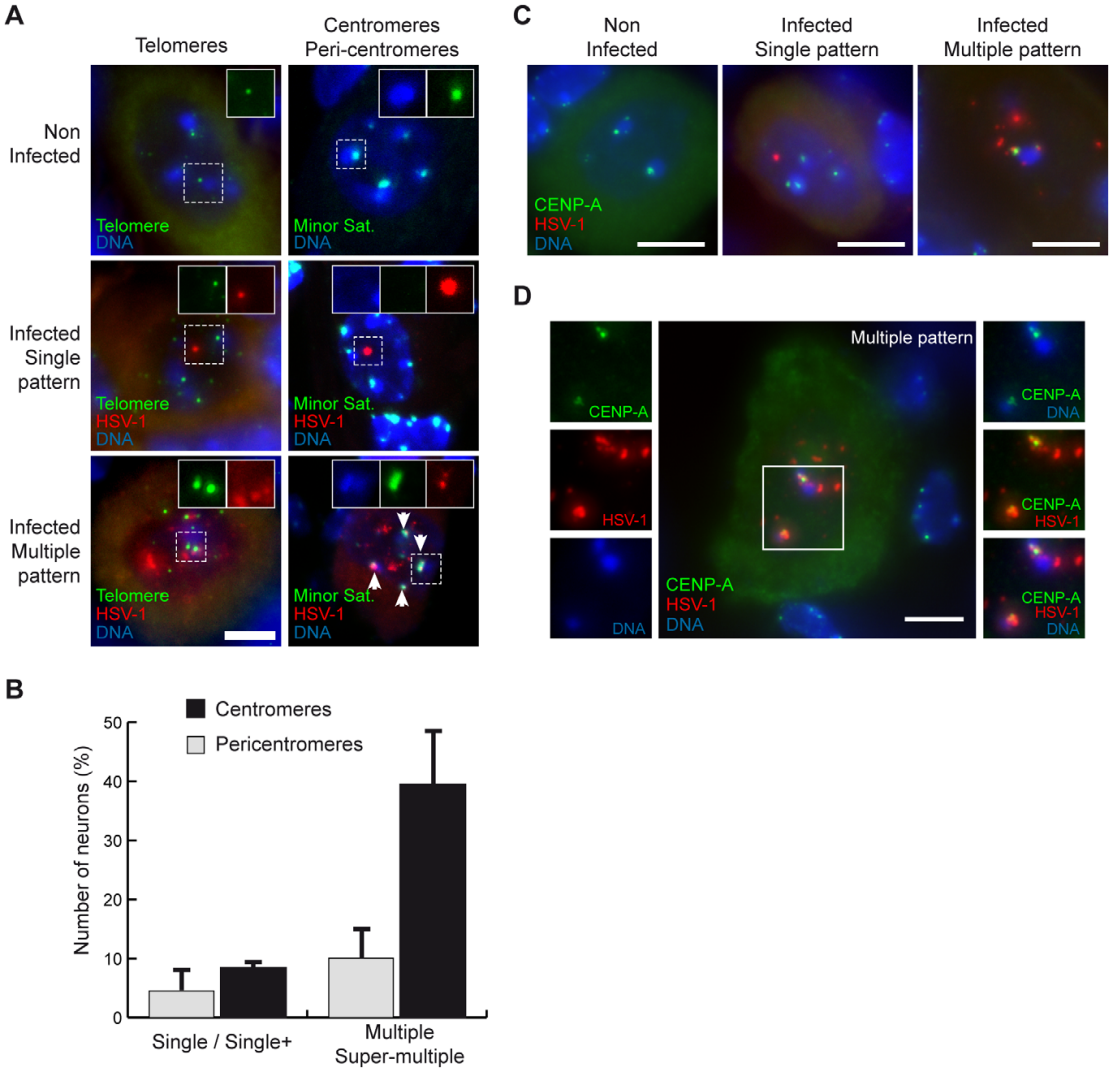
Latent HSV-1 genomes co-localize with centromeres in neuron nuclei. (A) Co-detection of HSV-1 genomes with telomeres and centromeres. TG sections from mock-infected and latently infected (SC16/lip) mice were stained by dual-color DNA-FISH using HSV-1-, telomere- and centromere-specific (Minor satellite, MiSat) probes. Tissues were counterstained with Hoechst 33258, which revealed aggregated pericentromeres in mouse cells (see text for details). The boxed areas show single-channel images. Arrowheads show co-localization of the HSV-1 signal with the minor satellite signal. Scale bar = 5 µm. (B) TG sections from two mice were stained with HSV-1 genome- and centromere-specific probes as in (A), and neurons in which the HSV-1 genome signal was associated with centromeres and pericentromeres were counted. Bars show the standard error of the mean. (C–D) TG sections from latently infected (SC16/lip) mice were stained by immuno-FISH with an HSV-1 genome-specific probe and anti-CENP-A antibody. Pericentromeres were counterstained with Hoechst. The images show that HSV-1 genome spots precisely co-localize with the CENP-A signal at the surface of pericentromeres. Scale bar = 5 µm.

The distribution of latent HSV-1 in neuron nuclei did not bear any resemblance to the patterns of known nuclear domains, and it remained unclear whether the viral genome associated with particular structures. As HSV-1 gene expression has been shown to involve viral chromatin [2], [3], we first focused on nuclear structures that are known to control cellular gene transcription through heterochromatin domains: the nuclear envelope, telomeres, centromeres, and pericentromeres. HSV-1 latent genomes were rarely found at the periphery of the nucleus, thus excluding a preferential association with the nuclear envelope. The association of the HSV-1 genome with telomeres and centromeres was assessed by dual-color DNA-FISH. No co-localization of the HSV-1 genome with telomeres was observed when assessing single or multiple patterns (Figure 3A). In mouse cells, the centromeres are positioned at the surface of pericentromeric aggregates (also called chromocenters), which are commonly detected by Hoechst staining [53] (Figure 3A and S2). By dual DNA-FISH, performed using specific probes to detect minor and major satellites, we confirmed that the organization observed in the cultured cells was similar to that in TG neurons, and that heterochromatic aggregates detected by DNA staining represent pericentromeres (Figure S2). Latent HSV-1 co-localized with centromeric repeats in 10.1±0.8% of the single-pattern neurons and in 39.4±8.8% of the multiple-pattern neurons (Figure 3B). In contrast, the frequency of association with pericentromeres remained low for both single- and multiple-pattern neurons (4.51±3.4% and 8.43±4.9%, respectively, *n* = 1,249 neurons in two mice). Within individual neuron nuclei, only a subset of HSV-1 FISH spots was associated with centromeres, showing that they are not the only residence sites of latent genomes. An immuno-FISH staining of the centromeric protein (CENP)-A, which is essential for the stability and functionality of the centromere [54] further confirmed the localization of a subset of HSV-1 genome onto the centromeres (Figure 3C–D). Additionally, these data demonstrate that the HSV-1-associated centromeric loci are likely to be functional centromeres. The association of HSV-1 genomes with centromeres did not appear to be an artifact caused by a strong HSV-1 signal in the multiple pattern for the following reasons: (i) the centromere and HSV-1 signals were largely co-localized (Figure 3A and 3D, bottom right image); (ii) the positioning of HSV-1 genomes adjacent to pericentromeres did not increase concomitantly with an increase in HSV-1 signal density (Figure 3B and S2); (iii) and co-detection of HSV-1 and telomeres did not result in signal co-localization, even though each cell contained twice as many telomeres as centromeres and telomeres are proximal to centromeres in acrocentric mouse chromosomes.

### Latent HSV-1 genomes associate with PML-NBs

**Figure 4:**
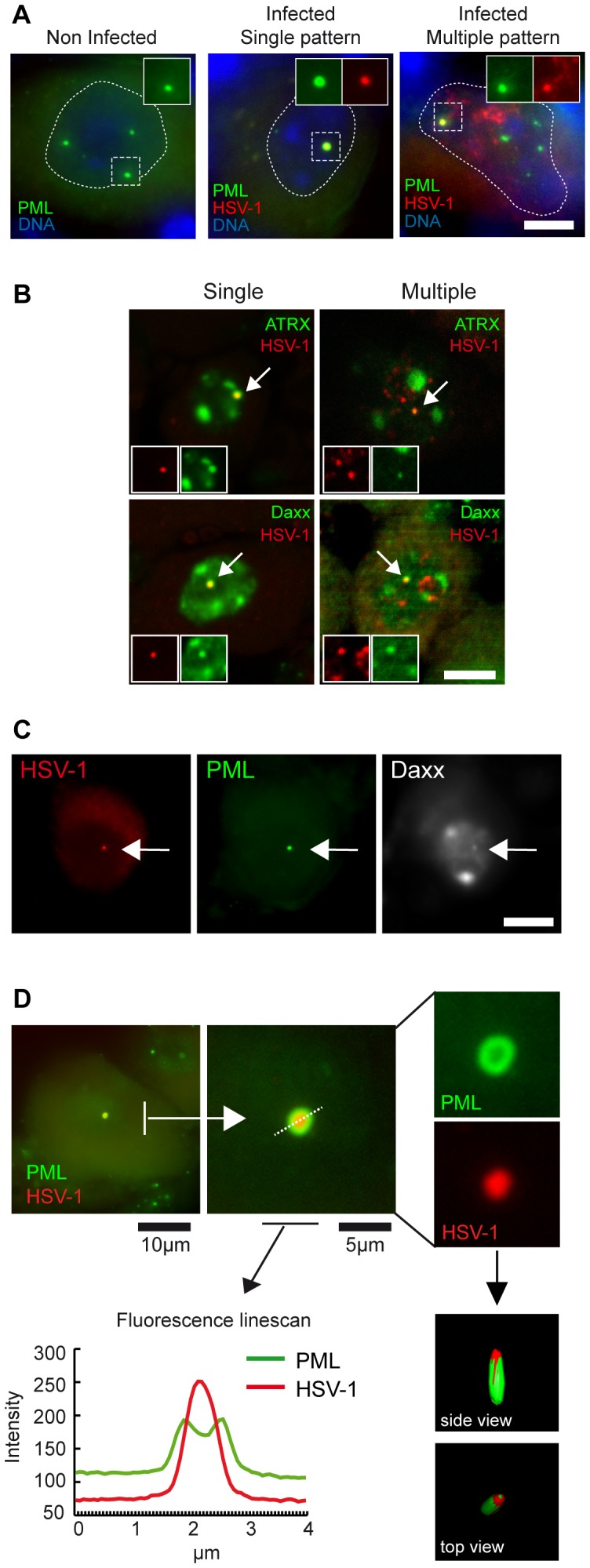
PML-NBs form around HSV-1 genomes in neurons. (A) Co-localization of HSV-1 genomes with PML protein. TG sections from mock-infected and latently infected (SC16/lip) mice (28 d.p.i.) were stained by immuno-FISH to co-detect the HSV-1 genome and PML protein. The boxed areas show single-channel images. Scale bar = 5 µm. (B) The HSV-1 genome associates with major PML-NB components. TG sections from latently infected mice were stained by immuno-DNA-FISH using anti-ATRX or anti-Daxx antibody and HSV-1 probe. HSV-1 genomes associated with ATRX or Daxx are indicated by arrows and enlarged in the insets. (C) TG sections from the same mouse as in (B) were stained by dual immuno-FISH using anti-PML and anti-Daxx antibodies and HSV-1 probe. The arrow shows an HSV-1-containing PML-NB stained with anti-Daxx antibody. (D) Confocal microscopic analysis of an HSV-1-containing PML-NB, showing (by fluorescence line-scan measurement and 3D reconstruction of a confocal Z-stack) that the HSV-1 DNA-FISH signal is localized within the PML-NB.

Because the single HSV-1 pattern did not frequently coincide with centromeres, and because a tight interplay between HSV-1 and PML-NBs exists *in vitro*, we developed an immuno-FISH approach to analyze whether PML-NBs could be involved in HSV-1 latency. In non-infected tissues, PML was detected by immuno-FISH in both non-neuronal cells and neurons. Neurons contained 1–10 PML spots, although a subpopulation of neurons did not display any detectable signal in the nucleus (Figure 4A). In mice at 28 d.p.i., a qualitative assessment of the number of PML-NBs in infected neurons did not reveal any obvious change, by comparison with uninfected neurons. In latently infected mice, PML protein invariably associated with single-pattern HSV-1 genomes, whereas it associated with HSV-1 genomes in only 61% of multiple-pattern neurons (*n* = 201 neurons). In the latter case, only some HSV-1 spots were associated with PML, revealing heterogeneity among the genomes regarding their association with PML.

**Figure 5:**
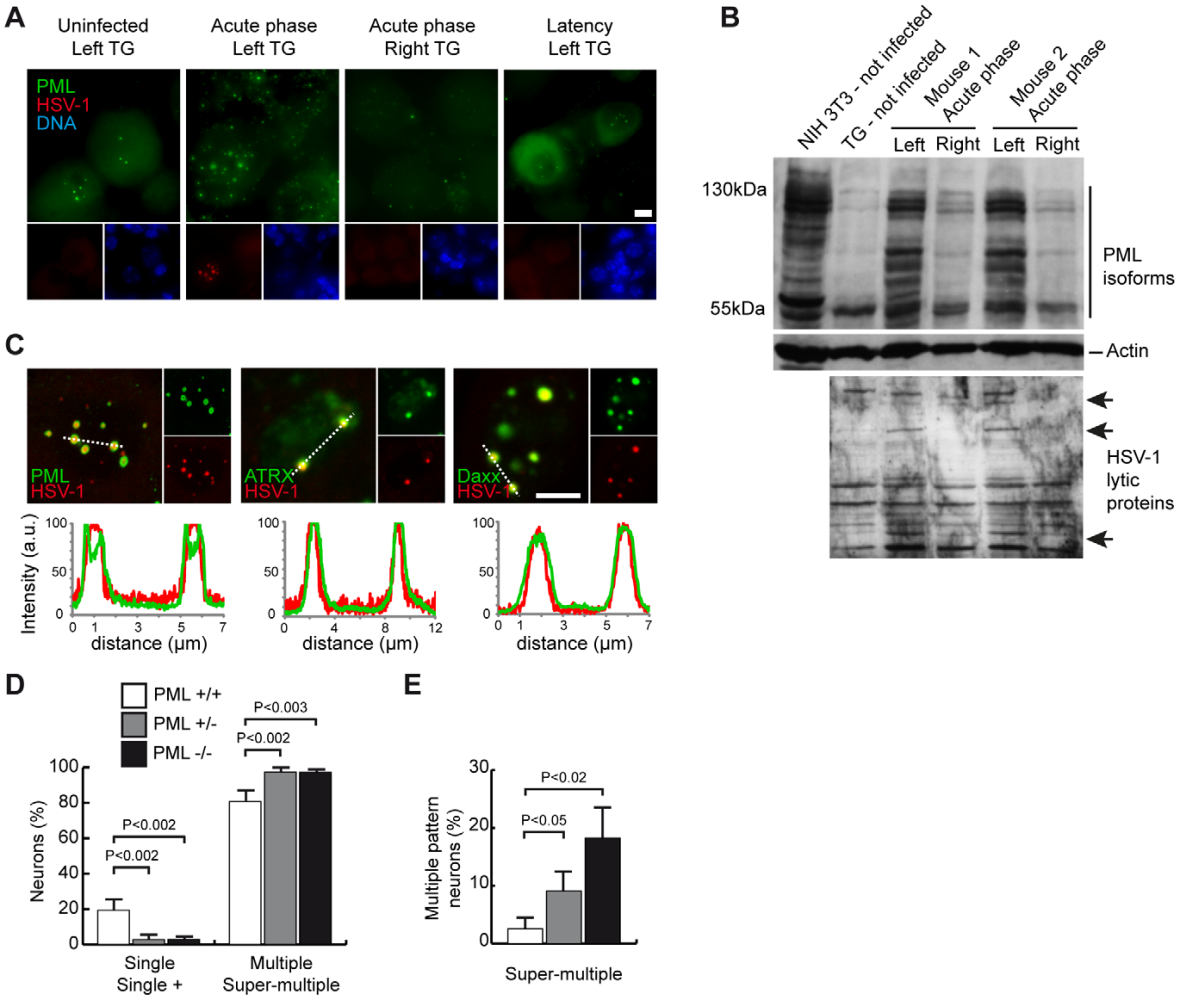
PML/PML-NBs control the distribution of incoming and latent HSV-1 genomes. (A–B) PML signals increase in neuronal and non-neuronal cells during the acute phase. (A) TG sections from mock-infected and infected mice sacrificed during the acute phase (6 d.p.i.) or latency (28 d.p.i.) were stained by immuno-FISH using HSV-1 probe and anti-PML antibody. In the SC16/lip model, inoculation of the virus on the upper left side induced asymmetrical acute and latent infection (see Materials and methods). All images were collected using identical gain and exposure settings. For acute infection, images of the left and right TG were collected from the same section. (B) Western blot analysis of PML protein in left and right TG harvested from mock-infected and infected mice during acute infection (6 d.p.i.). NIH3T3 cells were used as a positive control. A pan-HSV-1 serum was used to confirm ongoing acute infection in the left TG, through detection of lytic HSV-1 protein (arrows). Lytic infection is known to occur in early acute phase in some neurons and accessory cells, and is cleared as latency is being established [20], [107] and see figure S3). Actin was used as a loading control. (C) HSV-1-containing PML-NBs were detected during acute phase. TG sections from infected (SC16/lip) mice sacrificed at 6 d.p.i. were stained by immuno-FISH using HSV-1 probe and anti-PML (left), anti-ATRX (middle), and anti-Daxx (right) antibodies. The fluorescence plot profiles (dashed lines) are consistent with a ring-shaped PML-NB containing the HSV-1 genome and ATRX and Daxx proteins in its center. a.u. = arbitrary units. Scale bar = 5 µm. (D) The HSV-1 genome pattern is altered in PML-knockout mice. Wild-type, heterozygous, and PML-knockout mice were infected by corneal inoculation with the 17syn+ HSV-1 strain and were sacrificed at 28 d.p.i. HSV-1 genome patterns were quantified in serial sections spanning the whole ganglion. *n* = 4 mice per genotype (∼1500 infected neurons per genotype). Bars show the standard error of the mean. (E) Same data set as in (D). Fractions of neurons displaying very strong HSV-1 DNA-FISH signals (“super-multiple” pattern, Figure S1) within the multiple-pattern neurons in (D) are shown.

To determine whether HSV-1 genomes associated with *bona fide* PML-NBs, immuno-FISH and 3D microscopy were used to detect two stable signature components of PML-NBs, ATRX and Daxx. Both ATRX and Daxx were found to be associated with single-pattern HSV-1 genomes (Figure 4B); in multiple-pattern genomes, ATRX and Daxx co-localized with at least one HSV-1 genome focus, consistent with the observed frequency of the association of these genomes with PML. A triple-labeling experiment confirmed that PML and Daxx (Figure 4C) or PML and ATRX (not shown) simultaneously associated with the HSV-1 genome. Careful inspection of PML-NBs associated with HSV-1 revealed that PML protein had a ring-like shape, with HSV-1 genome in its center (Figure 4D). The presence of HSV-1 DNA within PML-NBs was intriguing because PML-NBs have been generally found to be devoid of nucleic acids and to be localized adjacent to or within 2 µm of genomic loci [37], [55]. High-resolution 3D confocal microscopy confirmed that in the case of the HSV-1 latent genome, the DNA was clearly inside the PML ring (Figure 4D), and that PML was wrapped around the viral genome. This organization was also observed during the early phase of mouse infection (Figure 5C) and *in vitro* in cells infected with replication-defective HSV-1 [50]. Thus, our observations show that PML assembles around HSV-1 genomic DNA, forming an atypical DNA-containing PML-NB (DCP-NB).

### Association of the HSV-1 genome with PML-NBs is initiated during early stages of the latency process and participates in HSV-1 genome pattern formation

PML-NB reorganization and co-localization with HSV-1 genomes were observed as early events in lytic infection in cultured cells [43], [45], [56], [57], raising the possibility that the association we observed during latency could be initiated early during the establishment of latency (the acute phase). In immuno-FISH analyzes performed on sections from mice sacrificed at 6 d.p.i., we observed that the PML protein signal within PML-NBs was stronger, and the PML-NBs were generally larger and more numerous in acute-phase tissues compared with latently infected and non-infected tissues (Figure 5A). Increases in the PML signal were observed in both neurons and accessory cells and, importantly, were restricted to infected TG (Figure 5A–B and S3; see Materials and methods for details), demonstrating that the increase in the PML signal resulted from the on-going infection. The increase in the PML signal could be attributable to the recruitment of nucleoplasmic PML (which accounts for 90% of nuclear PML; [36] into PML-NBs, or to an increase in the overall amount of PML. Western blotting of whole TG from mice at 6 d.p.i., showed increases in total PML and PML isoform levels in acutely infected TG compared with non-infected TG (see Materials and methods) and TG from non-infected mice (Figure 5B), demonstrating that the change in PML protein pattern results from an increase in total cellular PML protein and not only from a more efficient recruitment of nucleoplasmic PML into PML-NBs. These data support the stimulation of PML expression during the acute phase of HSV-1 infection, probably as a result of IFN pathway activation [41]. PML and HSV-1 formed 1–12 DCP-NBs per infected neuron nucleus, and most of them also contained ATRX, and Daxx (Figure 5C). Thus HSV-1 genome and PML patterns were significantly different from those observed in latently infected neurons. We conclude that acute infection provokes a PML response, leading to the formation of HSV-1 DCP-NBs, and that the association between PML and the viral genome is initiated during the very early stages of the latency process.

The above observations raised the possibility that the HSV-1/PML interaction may play a role in the formation of the latent HSV-1 patterns. To address this, we quantified HSV-1 latent genome patterns in latently infected PML-deficient mice. Both PML^+/−^ and PML^−/−^ mice displayed a significant decrease in the number of single-pattern neurons and a concomitant increase in the number of neurons with the super-multiple pattern (Figure 5D–E). These data show that PML protein and/or PML-NBs influence the intra-nuclear pattern adopted by the viral genome within latently infected neurons. Additionally, based on the number of viral genome foci detected within individual neurons, we conclude that in absence of PML/PML-NBs, the number of genome copy in latent TGs is higher, suggesting that PML/PML-NBs play a role in limiting the number of viral genomes that establish latency.

### Heterogeneity in HSV-1 genome transcriptional status in individual neurons

The above observations establish strong links between LAT expression, HSV-1 intra-nuclear distribution, and the association of the HSV-1 genome with PML-NBs and centromeres. This raised the possibility that the association of the HSV-1 genome with PML-NBs and centromeres may regulate LAT transcription. In support of this hypothesis, in single-pattern neurons, HSV-1 is systematically associated with PML-NBs, and LAT RNA is rarely present. To test whether association of genomes with PML NBs in multiple pattern cells had any effect on LAT expression in those cells, we performed a triple labeling experiment to simultaneously detect the HSV-1 genome, 2-kb LAT RNA, and PML/centromeres. Preliminary observations suggested that the 2-kb LAT signal was not correlated with the association of the HSV-1 genome with PML-NBs or centromeres. To confirm this, we traced LAT expression and the association of HSV-1 with PML and centromeres for each neuron across the entire TG of one mouse. The data clearly showed that there was no correlation (Table 2). This suggests that within a nucleus containing multiple copies of the HSV-1 genome, the association of some of the HSV-1 genomes with PML-NBs or centromeres does not have a dominant negative effect on the expression of LAT from the other copies of the viral genome.

**Table 2:** Correlation between PML-NBs or centromeres HSV-1 genome association and LAT expression.

Association with PML in multiple pattern neurons			Association with centromeres in multiple pattern neurons		
	LAT+	LAT−		LAT+	LAT−
**Associated**	33.7%	27.9%	**Associated**	27.0%	16.4%
**Not associated**	19.2%	19.2%	**Not associated**	26.0%	30.6%

To evaluate the impact of HSV-1 genome association with PML-NB (left) and centromeres (right), on LAT expression, LAT RNA and HSV-1 association with PML-NB or centromere was determined in individual neurons displaying multiple pattern of HSV-1 genome, using an immuno-RNA/DNA-FISH approach. The proportion of neurons in which an association of at least one HSV-1 spot and either PML or centromeres was determined in LAT+ and LAT− neurons. The values are in percentage of the total infected neurons within an entire TG of one latently infected mouse.

**Figure 6:**
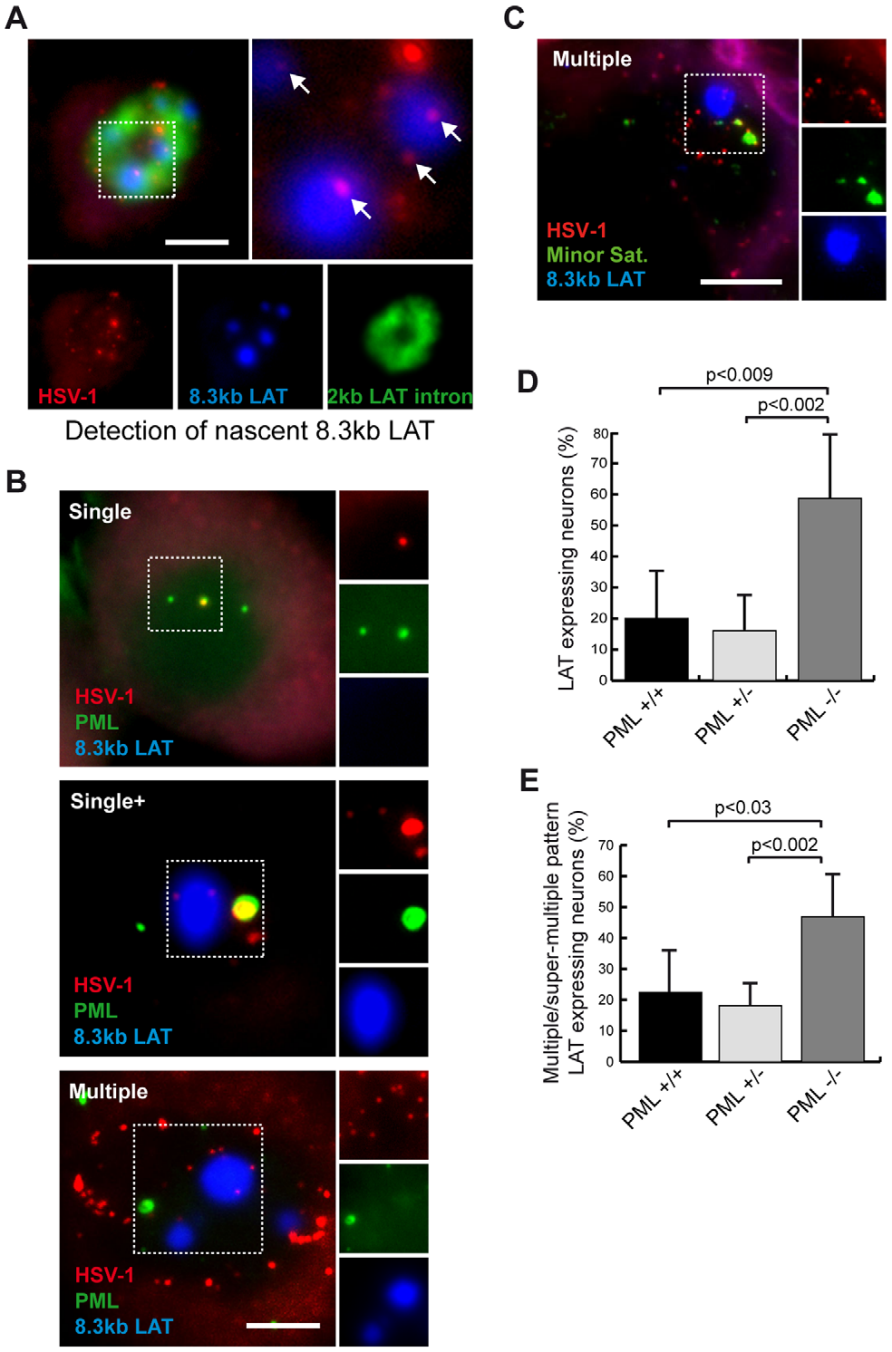
Absence of LAT transcription from PML-NB- and centromere-associated HSV-1 genomes. (A) Detection of nascent 8.3-kb primary LAT RNA. TG sections obtained from SC16/lip mice at 28 d.p.i. were stained by RNA/DNA-FISH using an HSV-1 genome DNA probe and LAT-2 (2-kb LAT) and LAT-5 (8.3-kb LAT) RNA probes (Figure 1B). (B) HSV-1 genomes producing nascent 8.3-kb LAT are not associated with PML-NBs. TG sections obtained from infected (SC16/lip) mice at 28 d.p.i. were subjected to dual immuno-FISH labeling with HSV-1 genome DNA probe, LAT-5 RNA probe, and anti-PML antibody. Examples of single, single+, and multiple patterns are shown. Dashed line images are single-channel images (right). Scale bar = 5 µm. (C) HSV-1 genomes producing nascent 8.3-kb LAT are not associated with centromeres. TG sections obtained from infected (SC16/lip) mice at 28 d.p.i. were subjected to dual RNA/DNA-FISH labeling with HSV-1 genome DNA probe, LAT-5 RNA probe, and minor satellite DNA probe. Scale bar = 5 µm. (D–E) PML wild-type, heterozygous, and knockout mice were infected (17syn+/eye) and sacrificed at 28 d.p.i. TG sections were stained by RNA/DNA-FISH using LAT-2 RNA probe and HSV-1 DNA probe. Graphs show (D) total numbers of neurons expressing 2-kb LAT RNA and (E) numbers of multiple/super multiple HSV-1 pattern neurons expressing 2-kb LAT RNA in PML^+/+^, PML^+/−^ and PML^−/−^ mice. *n* = 7 mice (∼2200 neurons) per genotype. Bars show the standard error of the mean.

An individual neuron contains a heterogeneous population of HSV-1 genomes (“free” or associated with a nuclear domain). Thus, the transcriptional status of these genomes may also be heterogeneous. To explore this possibility, we utilized the primary (nascent) 8.3-kb LAT transcript (Figure 1B) as a marker of the site of active transcription, in order to identify genomes that were being transcribed. Figure 6A illustrates the co-detection of HSV-1 genomes (red), the nascent 8.3-kb LAT transcript (blue), and the stable 2-kb LAT RNA (as a control). The nascent LAT RNA appeared as a set of large dots (1 to 7 per nucleus), each dot being associated with at least one HSV-1 genome spot (Figure 6). Such dotted pattern has been previously observed by ISH using peroxidase and alkaline phosphatase staining [58]. This suggests that in a single neuron, LAT can be transcribed from several copies of the HSV-1 genome. Notably, these neurons also contained several HSV-1 spots that were not associated with any 8.3-kb LAT RNA signal. Although we cannot exclude the possibility that these genomes are transcribed at a level below the sensitivity of our FISH method, the data suggest that they are not transcribed. Overall, these results show that only a fraction of the HSV-1 genomes within a single infected neuron are significantly transcribed and that the transcriptional status of HSV-1 genomes is highly heterogeneous in individual neurons.

### Absence of LAT transcription from PML-NB- and centromere-associated HSV-1 genomes

We next analyzed whether LAT is actively transcribed from PML-NB-associated latent HSV-1 genomes. In mouse TG sections, we detected the HSV-1 genome, its associated nascent LAT RNA product, and PML-NBs by a triple-labeling approach. In the LAT-expressing neurons, the nascent 8.3-kb LAT RNA was never associated with viral genomes that co-localized with PML-NBs (Figure 6B, bottom panel). We paid particular attention to 8.3-kb LAT-positive neurons with the single and single+ patterns, and observed that the LAT positive neurons were all neurons with a single+ pattern, and that the genome that was transcribed was the one not associated with PML. The larger HSV-1 genome spot surrounded by PML protein was never associated with an 8.3-kb LAT RNA spot (Figure 6B, top and middle panels). These data further support the idea that PML-NBs repress transcription of the associated HSV-1 genome. However, in the 8.3-kb LAT-positive neurons with the multiple pattern, several non-transcribed HSV-1 genomes were not associated with PML-NBs. It is likely that 8.3-kb LAT is not transcribed from many of the genomes and that factors other than PML-NBs also regulate LAT transcription.

We extended the analysis to centromere-associated HSV-1 genomes. Similarly to the findings for PML-NBs, centromere-associated viral genomes were never adjacent to the nascent 8.3-kb LAT RNA, suggesting that centromeres may also inhibit LAT transcription (Figure 6C).

If PML were to inhibit LAT transcription from the HSV-1 genome, one would expect to see an increase in LAT expression in a PML-deficient background. The 2-kb LAT RNA-FISH analysis of latently infected PML^+/−^ and PML^−/−^ mice revealed that the percentage of 2-kb LAT-positive neurons was higher in PML^−/−^ mice compared with wild-type and heterozygous mice (Figure 6D). The increase in the LAT-positive neuron percentage was not simply related to greater numbers of neurons with multiple/super-multiple pattern. Indeed, within this category of neurons, LAT expression was twofold higher in PML^−/−^ animals (Figure 6E). These data demonstrate that PML/PML-NBs play a role in the regulation of LAT expression and support their transcriptional repressor activity.

## Discussion

Here, we report the structural and functional interactions of the genomes of a persistent virus, HSV-1, with the host-cell nuclear environment. Our data reveal two new features of the viral genomes that characterize the latency state. First, the intra-nuclear distribution of the latent genome is not random and correlates with viral gene expression, and second, the host-cell nuclear domains play a role in viral genome pattern acquisition and in the control of viral gene expression. Thus, the interaction between the viral genomes and host-cell nuclear components represents a new level of host–virus interaction, which is likely to participate in the process of latency and reactivation.

The ability to explore the cell host–virus interaction is of outmost importance in our understanding of persistent viral infections because regulation of the latent HSV-1 genome relies mainly on cellular components. A substantial benefit of the in situ immuno-DNA/RNA-FISH developed in this study lies in the simultaneous detection of the viral genome, virally encoded transcripts, and cellular components in the same cell. This will enable us to address important issues of cell host–virus interactions in tissues obtained from physiologically infected animal models but also in emerging in vitro HSV-1 latency models [59]. The FISH approach provides high-resolution individual cell data without sacrificing the access to a global view of the virus and of the host-cell population. This appears as a major advantage given that HSV-1 latency is highly heterogeneous. FISH and immuno-FISH will be essential assets to study latency and will complement the currently used biochemical and molecular approaches.

We confirmed that LAT was expressed in a fraction of infected neurons and that viral copy numbers varied among neurons. Based on the HSV-1 genome pattern and our estimate of genome copy number per FISH spot, the single and multiple patterns likely represent the low-copy and high-copy virus genome-containing neurons, respectively, identified by contextual analysis [19]. Additionally, we found that the HSV-1 latent genomes were heterogeneously distributed within neuron nuclei and preferentially associated with PML-NBs and centromeres. LAT expression is positively correlated with HSV-1 genome pattern and negatively correlated with its association with PML-NBs and centromeres, demonstrating that the intra-nuclear distribution of HSV-1 genomes is a major feature of the latency process. LAT detection was almost exclusively associated with the multiple genome pattern, demonstrating that LAT expression is restricted to neurons with high viral genome copy numbers. Single-cell contextual analysis has also revealed that a high genome copy number per neuron is associated with a higher probability of virus reactivation [14], suggesting that this parameter may be a key aspect of latent genome status. Importantly, the various copies of HSV-1 within a single nucleus are not transcriptionally equivalent, with LAT being transcribed only from a subset of genomes. This suggests that latent HSV-1 genomes are comparable to, and behave like, multi-allelic cellular genes, raising the possibility that only a subset of these genomes are susceptible to sustain full reactivation (i.e., to reach expression of lytic genes). The dotted pattern observed with the 8.3 kb LAT probe was previously reported [58], and was proposed to be sites of early processing of the LAT transcript. Our data confirm this hypothesis by demonstrating that these clouds of LAT primary RNA are associated with HSV-1 genomes.

PML-NBs are probably the most thoroughly studied nuclear domains in the context of virus infection for their involvement in the innate antiviral response and in the interferon (IFN) response pathway. Our data from the acute phase and from PML^−/−^ mice support a role for PML-NBs in limiting the extent of viral replication during acute-phase, and thus the number of HSV-1 genomes that establish latency in each neuron. These data are consistent with the known role of PML-NBs, through the activity of several of their major components such as PML, Sp100, Daxx, ATRX, and small ubiquitin-like modifier (SUMO) protein, as repressors of HSV-1 onset of lytic infection in cultured cells [57], [60]–[63]. We provide a clear demonstration that PML expression increased in vivo in acutely infected mouse TG, and that the PML protein, through the formation of HSV-1-containing PML-NBs, associated with the HSV-1 genome during the early phase of latency. Additionally, we showed that the HSV-1 genomes remain associated with PML-NBs during latency in over 80% of infected neurons, suggesting that PML-NBs play an antiviral role influencing latency and probably reactivation.

PML-NBs have been proposed to create a specific local nuclear environment by concentrating proteins and hosting biochemical reactions within the PML shell. PML-NBs reorganize in response to various stressors, potentially to relocate their activity at selected nuclear sites [64]. The reorganization of the PML-NBs resulted in the formation of new PML-NBs around the HSV-1 genome at early stages of latency, thus altering the immediate nuclear environment of the incoming viral genome. Importantly, we showed that PML-NBs remain associated with viral genomes long after replicative infection has ceased, indicating that maintenance of this particular type of DNA-containing PML-NB (DCP-NB) requires neither on-going viral replication nor the associated antiviral and IFN signaling pathways. The pattern of both HSV-1 genome and PML-NBs are dramatically different between acute phase and latency, indicating that a profound remodeling of these patterns takes place during establishment of latency. Ongoing studies will provide pattern analysis at intermediate time between 6 d.p.i. and 28 d.p.i. The formation of DCP-NBs can be seen as a response to the presence of chromatinized foreign DNA [42], or more broadly, the presence of pathology-associated abnormal chromatin [65], [66]. Moreover, PML-NBs repress the synthesis of the LAT primary transcript through their association with HSV-1 genomes, from which microRNAs are produced [10]. The atypical assembly of a PML-NB around a genetic locus may thus be considered a distinct form of PML-NB controlling the expression of noncoding RNA in pathological situations. Indeed, PML-NBs assemble around pericentromeric satellite sequences and telomeres, two cellular loci known to give rise to noncoding RNA [65], [66].

We showed that HSV-1 genomes are also associated with host neuron centromeres during latency. Bishop and colleagues previously showed that foreign DNA delivered by polyomavirus-like particles was localized to centromeres [42]. Our data from a biologically relevant context and an *in vivo* model support the idea that centromeres represent docking sites for virus genomes. Centromeres and the adjacent pericentromeres are among the best-characterized nuclear domains that silence nearby genes [29]–[31]. Consistent with this, we showed that centromere-associated HSV-1 genomes did not express LAT RNA. We want to emphasize that the association with HSV-1 occurs at the centromere itself, which distinguishes the current set of data from most other published data related to associations of cellular genes with pericentromeres [67]–[75]. Only a subset of HSV-1 genomes within a nucleus is found associated with centromeres, indicating that this association is not the main mechanism repressing transcription of latent genomes. Of note, both PML-NBs and centromeres (because of their proximity with pericentromeres) are enriched in ATRX and Daxx. In addition, hDaxx has been shown to co-localize with centromeres in human cells [76]. This raises the possibility that both nuclear domains exert their repressive effect on HSV-1 transcription through common factors [62].

Interestingly, HSV-1 has developed strong “anti-centromere” activity through the combined activities of the viral E3 ubiquitin ligase ICP0 protein [77] and the proteasome. In cultured cells, ICP0 induces the degradation of at least 10 CENPs, which results in the alteration of centromeric chromatin and destabilization of the centromeres [78]–[81] (S. Gross and P. Lomonte, personal communication). The biology of HSV-1 does not favor ICP0-induced centromere destabilization, prompting the mitotic arrest of infected cells [82], [83]. Indeed, HSV-1 is able to replicate independently of the cell cycle [84], and the lytic cycle does not depend on cell arrest at the mitotic phase. This suggests that HSV-1 targets centromeres not to control their effect on chromosome segregation, but rather to control an activity more relevant of differentiated, non-dividing cells. On the other hand, it is suspected that ICP0, which does not bind DNA and is not a transcription factor *per se* [85], inhibits the activities of numerous repressive nuclear factors in order to favorably modify the nuclear environment to stimulate the virus replicative cycle, at both the onset of a new infection and during the course of reactivation [60], [86]–[93]. ICP0 is known to be essential for full reactivation of HSV-1 in latently infected quiescent cells [94]–[96]. We therefore propose that centromeres, although they seem to act as repressors during latency, may offer a favorable nuclear environment for transcriptional events during reactivation, providing their protein composition and structure are modified by ICP0. This agrees with data showing that centromere/pericentromere regions are sites of intense transcriptional activity following the exposure of cells to a variety of stressors such as heat shock, UV, and heavy metals, which potentially induce HSV-1 reactivation [97]–[99]. On-going work should provide evidence to support this hypothesis.

## Materials and Methods

### Ethics statement

For animal experiments performed in France: all procedures involving experimental animals conformed to ethical issues from the Association for Research in Vision and Ophthalmology (ARVO) Statement for the use of animals in research, and were approved by the local Ethical Committee of UPR-3296-CNRS, in accordance with European Community Council Directive 86/609/EEC. All animals received unlimited access to food and water.

For animal experiments performed in the USA: animals were housed in American Association for Laboratory Animal Care-approved housing with unlimited access to food and water. All procedures involving animals were approved by the Children's Hospital Animal Care and Use Committee and were in compliance with the Guide for the Care and Use of Laboratory Animals.

### Virus strains, mice and virus inoculation

Wild-type HSV-1 strains SC16 and 17syn+ were used. Stocks were generated in rabbit skin cell monolayers, and viral titers were determined as described previously [100]. Briefly, six-week-old inbred female BALB/c mice (Janvier Breeding, Le Genest Saint Ile, France), were inoculated with 10^6^ PFU of the SC16 virus, injected into the upper-left lip of the mice. Mice were observed daily for clinical signs of ocular infection from 0 to 28 d.p.i. The sided inoculation of the lip results in an asymmetrical infection, which is characterized by an extremely low load of virus on the right TG compared to the left TG. Thus, data presented in this study were collected on the left TG, except in Figure 3A and 3D, as indicated [4]. Data presented in this study were collected from the left TG, except for those shown in Figure 3A and 3D. For the 17syn+/eye model, inoculation was performed as described previously [101]. Briefly, prior to inoculation, mice were anesthetized by intra-peritoneal injection of sodium pentobarbital (50 mg/kg of body weight). A 10 µL drop of inoculum containing 10^5^ PFU of 17syn+ was placed onto each scarified corneal surface. This procedure results in ∼80% mice survival and 100% infected TG. PML wild-type, heterozygous, and knockout mice were obtained from the NCI Mouse Repository (NIH, http://mouse.ncifcrf.gov; strain, 129/Sv-*Pml^tm1Ppp^*) [102]. Genotypes were confirmed by PCR, according to the NCI Mouse Repository guidelines.

Primers:

P009: 5′-cTG cGc TGc ccG AGc TGc cAG G -3′
P010: 5′-cAG cGc AGG GTT GcG GTG GTT GG -3′
P011: 5′-cTc ccG ATT cGc AGc GcA TcG cc -3′

### Frozen sections

Frozen sections of mouse TG were performed as previously described [100]. Mice were anesthetized at 6 or 28 d.p.i., and before tissue dissection, mice were perfused intra-cardially with a solution of 4% formaldehyde, 20% sucrose in 1× PBS. The whole head, or individual TG were prepared as previously described, and 10 µm frontal sections were collected in three parallel series, and stored at −80°C.

### DNA-FISH

DNA-FISH probes were Cy3 labeled by nick-translation as described previously [103]. Briefly, cosmids 14, 28 and 56 [104] comprising a total of ∼90 kb of HSV-1 genome (see Figure 1A) were labeled by Nick translation (Roche Diagnostic) with dCTP-Cy3 (GE Healthcare), and stored in 100% formamide (Sigma-Aldrich). The DNA-FISH procedure was adapted from Solovei et al. [105].

Frozen sections stored at −80°C were thawed, rehydrated in 1× PBS and permeabilized in 0,5% Triton X-100. Heat based unmasking was performed in 100 mM citrate buffer, and sections were post-fixed using a standard methanol/acetic acid procedure, and dried for 10 min at RT. DNA denaturation of section and probe was performed for 5 min at 80°C, and hybridization was carried out overnight at 37°C. Hybridization mix contained 30 ng of each probe in 10% dextran, 1× denhardt, 2XSSC, 50% formamide. Sections were washed 3×10 min in 2XSSC and 3×10 min in 0.2XSSC at 37°C, and nuclei were stained with Hoechst 33258 or ToPro3 (Invitrogen). All sections were mounted under coverslip using Vectashield mounting medium (Vector Laboratories) and stored at +4°C until observation.

### Immuno-DNA-FISH

Frozen sections were treated as described for DNA-FISH up to the antigen-unmasking step. Tissues were then incubated for 24 h with the primary antibody (diluted at 1/100). After three washes, secondary antibody (1/200) was applied for 1 h. The secondary antibodies (Invitrogen) were either AlexaFluor-conjugated (PML, CENP-A), or HRP conjugated (ATRX and Daxx), which were subsequently detected by enzymatic amplification according to manufacturer's guideline (TSA, Invitrogen). Following immunostaining, the tissues were post-fixed in 1% PFA, and DNA-FISH was carried out from the methanol/acetic acid step onward.

### Dual RNA/DNA-FISH

RNA-FISH probe labeling and RNA-FISH procedures were performed as described previously [4]. Biotinylated single-strand RNA probes were prepared by *in vitro* transcription (Ambion) using plasmids pSLAT-2, pSLAT-4 and pSLAT-6 as template (see Figure 1) (Kind gift of S. Efstathiou, University of Cambridge, UK). Frozen sections were treated as described for DNA-FISH up to the antigen-unmasking step using solutions containing 2 mM the RNAse inhibitor Ribonucleoside vanadyl complex. The sections were pre-hybridized in 50% formamide/2× SSC and hybridized overnight at 65°C with 50 ng of RNA probe a 50% formamide buffer. Sections were washed in50% formamide/2× SSC at 65°C, and in 2× SSC at room temperature. Detection was performed using streptavidin-HRP conjugate, followed by TSA amplification (Invitrogen) with an AlexaFluor 350 conjugated substrate, according to the manufacturer's guidelines. The DNA-FISH procedure was performed starting from the methanol/acetic acid post-fixation step.

### Antibodies

The following primary antibodies were used: anti-mouse PML (mAb3739, Millipore), anti-mouse CENP-A (rabbit mAb C51A7, Cell Signaling Technologies), anti-ATRX H-300 (Santa Cruz Biotechnology), anti-Daxx M-112 (Santa Cruz Biotechnology), anti-pan-HSV-1 (LSBio), and anti-mouse actin (Sigma-Aldrich). All secondary antibodies were Alexa Fluor-coupled and were raised in goats (Invitrogen). HRP-coupled secondary antibodies were provided with the TSA kit (Invitrogen).

### Microscopy and imaging

Observations and most image collections were performed using an inverted Cell Observer microscope (Zeiss) with a Plan-Apochromat ×100 N.A. 1.4 objective and a CoolSnap HQ2 camera from Molecular Dynamics (Ropper Scientific). When indicated, images were collected on a Zeiss LSM 510 confocal microscope using a Plan-Apochromat ×63 N.A. 1.4 objective, except for those shown in Figure 2E, which were collected using a Zeiss LSM 780 microscope. Line scans, 3D projections, and surface rendering were performed using AIM and Zen software (Zeiss).

### Western blotting

TGs were collected at 6 or 28 d.p.i. and snap-frozen. Frozen tissues were ground, thawed in lysis buffer (10 mM Tris-EDTA, pH 8.0) containing a protease inhibitor cocktail, and briefly sonicated. Protein extracts were homogenized using QiaShredders (Qiagen). Protein concentration was estimated by the Bradford method. Extracted proteins were analyzed by Western blotting using anti-mouse PML antibody (mAb3739, Millipore) [106].

## Supporting Information

Figure S1 *In situ* detection of HSV-1 genome by DNA-FISH on mouse TG sections. (A) Control experiment demonstrating the specificity of the HSV-1 DNA-FISH signal. TG section of 28 d.p.i. infected mice (SC16/lip infection), were stained by dual color DNA-FISH using the Cy3 labeled Cos64 control probe (Cos64 Cy3 probe) or a mix of HSV-1 Cos14, Cos28 and Cos56 probes (HSV-1 Cy3 probe) and a commercially available biotinylated HSV-1 probe (Enzo Life Sciences). The Cos64 probe was prepared from the empty cosmid backbone present in the Cos14, Cos28 and Cos56 vectors. The biotinylated HSV-1 probe was detected using TSA technology (Invitrogen). (B) Images of neurons containing 2 types of underrepresented HSV-1 genome patterns. Same experiment as in figure 1D. The “single+” pattern contains one spot similar to the spot of the “single” pattern, and an additional 1 or 2 smaller spots. The “super-multiple” pattern corresponds to neurons containing a large amount of viral DNA that fills the entire nucleus, as numerous spots or very large aggregates (4 µm or more). Such pattern is rarely observed in the SC16/lip inoculation model, and is present in a few percent of neurons in the 17syn+/eye inoculation model (see Figure 5E). Scale = 5 µm.
(TIF)

Figure S2 Specific localization of HSV-1 latent genomes on the centromeres at the surface of pericentromeres. (A) Intranuclear organization of centromeres and pericentromeres in mouse neuronal tissues. Dual color DNA-FISH was performed on TG section of a non-infected mouse, using a biotinylated Minor satellite probe (revealed with AlexaFluor 488 conjugated streptavidin) and a Cy3 labeled Major satellite probe. Pericentromeres (major sat.) of several chromosomes aggregate into 2 to 5 clusters that are frequently found next to the nucleolus. Centromeres (minor sat.) are located at the surface of the pericentromeres. This organization is similar to what has been described in cultured cells [108], and demonstrates that DNA staining by Hoechst is a relevant approach for the detection of pericentromeres. The number and organization of pericentromeric aggregates are consistent with previous studies [109]. (B) Colocalization of HSV-1 genomes with centromeres in multiple pattern is not due to high density of HSV-1 genome spots. Same experiment and data set that are presented in figure 3B. We re-analyzed the data and sub-divided the “multiple pattern” neurons into 2 categories: neurons with distinct spots of 1–2 µm in diameter (example: multiple pattern in figure 1D), and neurons with large spots and/or a cloud of fine spots (example: multiple pattern in figure 3A). The results showed that the denser the HSV-1 spots, the higher the localization at centromeres. However, the association with pericentromeres remained low even in nuclei harboring an abundant HSV-1 signal. Data are from 3 mice (1865 neurons).
(TIF)

Figure S3 PML and PML-NBs abundance is related to ongoing acute infection. (A) Asymmetrical acute infection in the SC16/lip model. HSV-1 DNA-FISH was performed on sections of a 6 d.p.i. infected mouse using the HSV-1 Cy3 labeled probe. The section was imaged with a 40× objective on a widefield microscope using a tiling scan module. At this magnification, the high auto-fluorescence of the tissue provides a map of the TGs. A close up view of the left TG reveals neurons in which FISH signal is very high, and marks ongoing acute infection. Scale = 100 µm. (B) Asymmetrical increase of PML and PML-NB signal during acute infection. Same experiment as in figure 5A. Shown are low magnification images of the left and right TG of an acutely infected mouse, after anti-PML immunofluorescence. Scale = 50 µm.
(TIF)
