# Supplementary figures and images for: HSV-1 Genome Subnuclear Positioning and Associations with Host-Cell PML-NBs and Centromeres Regulate LAT Locus Transcription during Latency in Neurons
Source: PLoS Pathog. 2012 Aug 9;8(8):e1002852. doi: 10.1371/journal.ppat.1002852 (PMC3415458; doi:10.1371/journal.ppat.1002852)

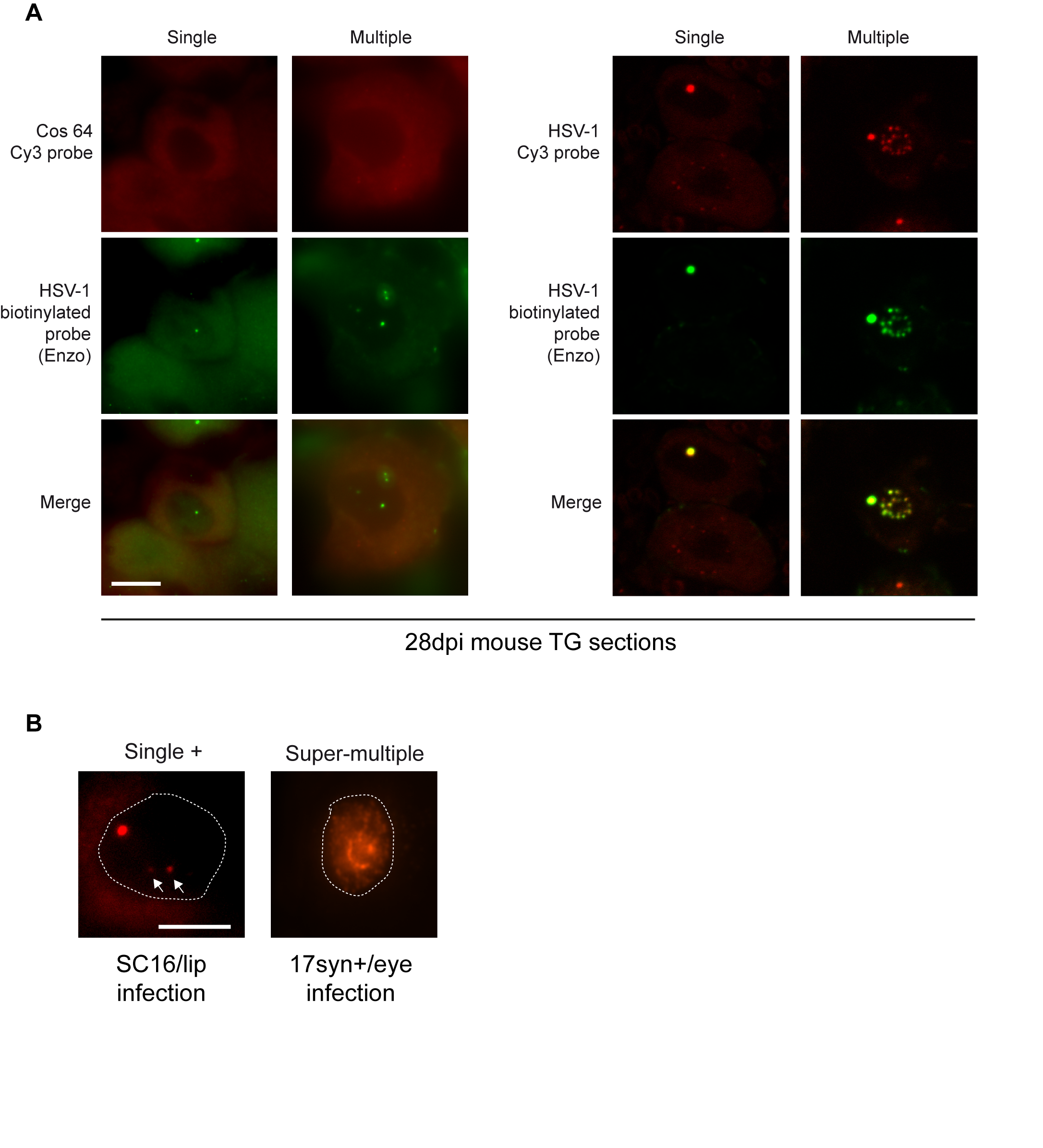

Supplement: Figure S1 — In situ detection of HSV-1 genome by DNA-FISH on mouse TG sections. (A) Control experiment demonstrating the specificity of the HSV-1 DNA-FISH signal. TG section of 28 d.p.i. infected mice (SC16/lip infection), were stained by dual color DNA-FISH using the Cy3 labeled Cos64 control probe (Cos64 Cy3 probe) or a mix of HSV-1 Cos14, Cos28 and Cos56 probes (HSV-1 Cy3 probe) and a commercially available biotinylated HSV-1 probe (Enzo Life Sciences). The Cos64 probe was prepared from the empty cosmid backbone present in the Cos14, Cos28 and Cos56 vectors. The biotinylated HSV-1 probe was detected using TSA technology (Invitrogen). (B) Images of neurons containing 2 types of underrepresented HSV-1 genome patterns. Same experiment as in figure 1D. The “single+” pattern contains one spot similar to the spot of the “single” pattern, and an additional 1 or 2 smaller spots. The “super-multiple” pattern corresponds to neurons containing a large amount of viral DNA that fills the entire nucleus, as numerous spots or very large aggregates (4 µm or more). Such pattern is rarely observed in the SC16/lip inoculation model, and is present in a few percent of neurons in the 17syn+/eye inoculation model (see Figure 5E). Scale = 5 µm. (TIF) [file ppat.1002852.s001.tif]

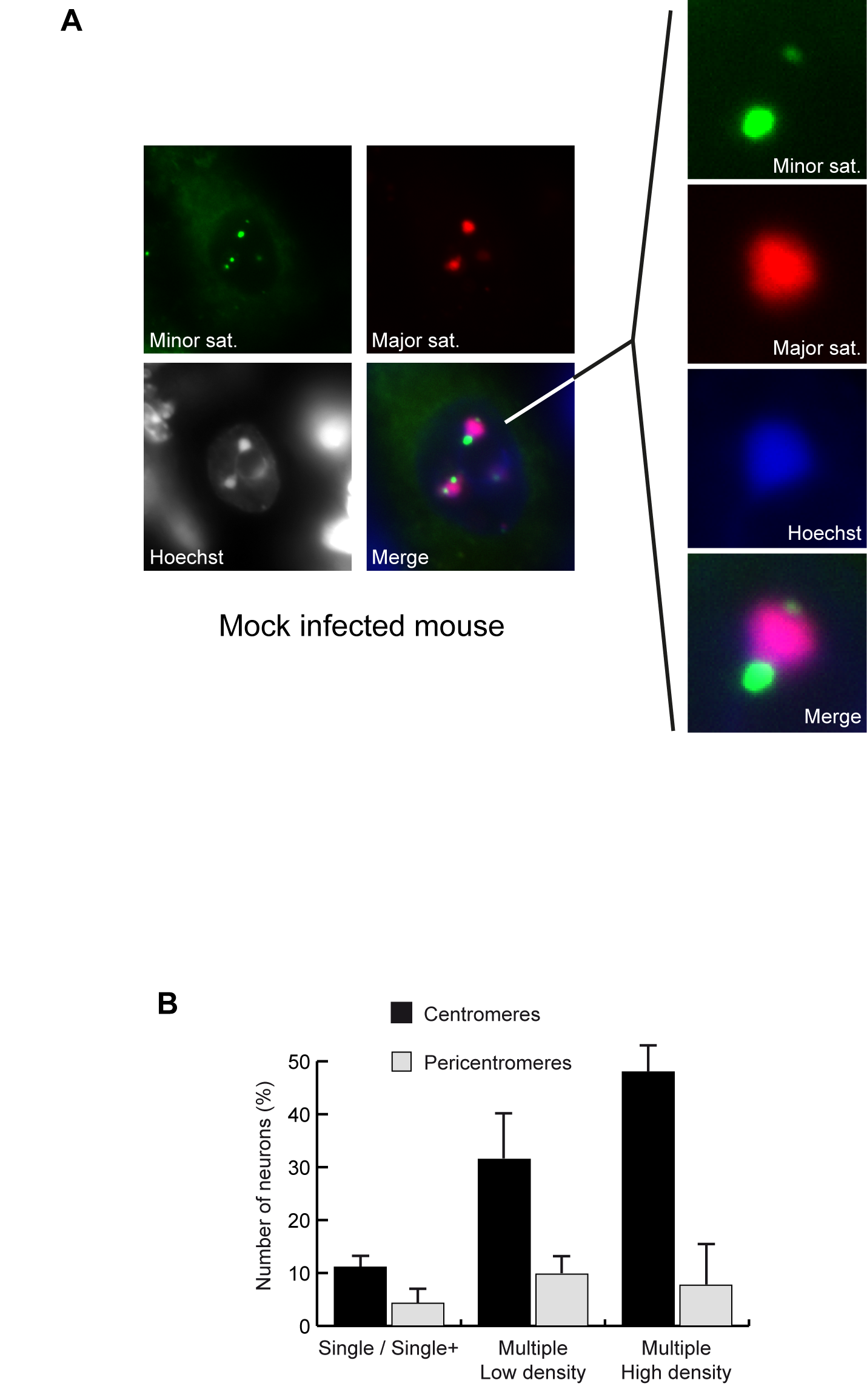

Supplement: Figure S2 — Specific localization of HSV-1 latent genomes on the centromeres at the surface of pericentromeres. (A) Intranuclear organization of centromeres and pericentromeres in mouse neuronal tissues. Dual color DNA-FISH was performed on TG section of a non-infected mouse, using a biotinylated Minor satellite probe (revealed with AlexaFluor 488 conjugated streptavidin) and a Cy3 labeled Major satellite probe. Pericentromeres (major sat.) of several chromosomes aggregate into 2 to 5 clusters that are frequently found next to the nucleolus. Centromeres (minor sat.) are located at the surface of the pericentromeres. This organization is similar to what has been described in cultured cells [108], and demonstrates that DNA staining by Hoechst is a relevant approach for the detection of pericentromeres. The number and organization of pericentromeric aggregates are consistent with previous studies [109]. (B) Colocalization of HSV-1 genomes with centromeres in multiple pattern is not due to high density of HSV-1 genome spots. Same experiment and data set that are presented in figure 3B. We re-analyzed the data and sub-divided the “multiple pattern” neurons into 2 categories: neurons with distinct spots of 1–2 µm in diameter (example: multiple pattern in figure 1D), and neurons with large spots and/or a cloud of fine spots (example: multiple pattern in figure 3A). The results showed that the denser the HSV-1 spots, the higher the localization at centromeres. However, the association with pericentromeres remained low even in nuclei harboring an abundant HSV-1 signal. Data are from 3 mice (1865 neurons). (TIF) [file ppat.1002852.s002.tif]

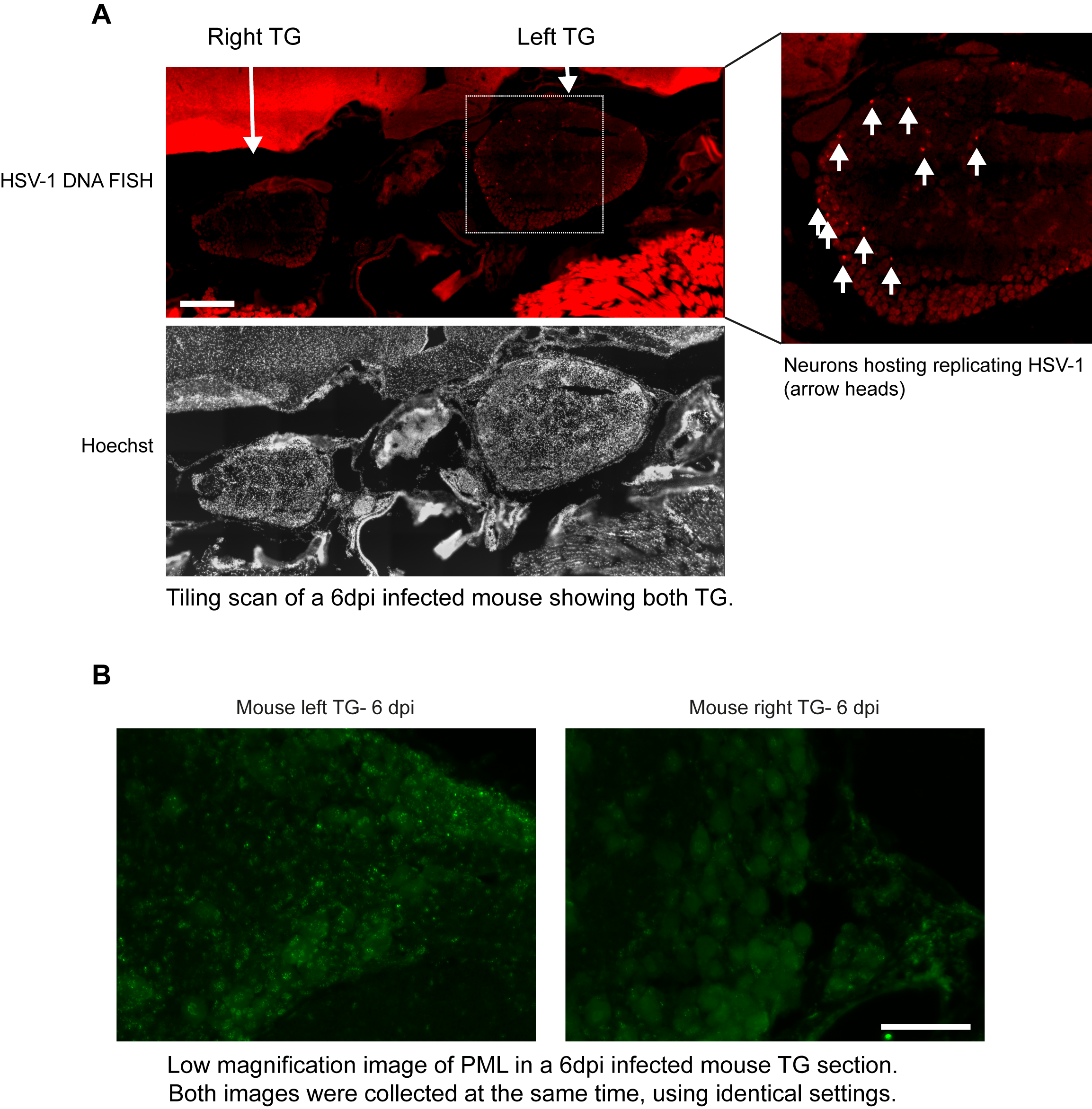

Supplement: Figure S3 — PML and PML-NBs abundance is related to ongoing acute infection. (A) Asymmetrical acute infection in the SC16/lip model. HSV-1 DNA-FISH was performed on sections of a 6 d.p.i. infected mouse using the HSV-1 Cy3 labeled probe. The section was imaged with a 40× objective on a widefield microscope using a tiling scan module. At this magnification, the high auto-fluorescence of the tissue provides a map of the TGs. A close up view of the left TG reveals neurons in which FISH signal is very high, and marks ongoing acute infection. Scale = 100 µm. (B) Asymmetrical increase of PML and PML-NB signal during acute infection. Same experiment as in figure 5A. Shown are low magnification images of the left and right TG of an acutely infected mouse, after anti-PML immunofluorescence. Scale = 50 µm. (TIF) [file ppat.1002852.s003.tif]
